# Prothrombin complex concentrate in surgical patients: retrospective evaluation of vitamin K antagonist reversal and treatment of severe bleeding

**DOI:** 10.1186/cc8186

**Published:** 2009-11-30

**Authors:** Kerstin S Schick, Jan M Fertmann, Karl-Walter Jauch, Johannes N Hoffmann

**Affiliations:** 1 Department of Surgery, University of Munich - Großhadern, Marchioninistrasse 15 81377 Munich, Germany

## Abstract

**Introduction:**

Prothrombin complex concentrates are recommended for rapid reversal of vitamin K anticoagulants. As they normalize levels of vitamin K dependent clotting factors and re-establish hemostasis, they may also be used as adjunctive therapy in patients with major bleeding. The aim of this study was to retrospectively evaluate the efficacy of prothrombin complex concentrates in the surgical setting.

**Methods:**

The case notes of 50 patients requiring urgent oral anticoagulation reversal (n = 12) or with severe perioperative coagulopathic bleeding (n = 38) who received an infusion of prothrombin complex concentrate (Beriplex P/N(R) 500) at the surgical department of the University of Munich Hospital, Germany were retrospectively reviewed. Efficacy of prothrombin complex concentrate application was evaluated using the Quick test, reported as an international normalized ratio, hemodynamic measurements and requirement for blood products. Safety assessments included whole blood hemoglobin levels and specific parameters of organ dysfunction.

**Results:**

Baseline characteristics were comparable, except that mean baseline international normalized ratio and hemoglobin levels were significantly higher (*P *< 0.01) in anticoagulation reversal than in bleeding patients. In anticoagulation reversal, the international normalized ratio was significantly reduced (from 2.8 +/- 0.2 at baseline to 1.5 +/- 0.1, *P *< 0.001) after one prothrombin complex concentrate infusion (median dose 1500 IU; lower quartile 1,000, upper quartile 2,000). No major bleeding was observed during surgery after prothrombin complex concentrate administration. Only one patient received platelets and red blood cell transfusion after prothrombin complex concentrate administration. In bleeding patients, infusion of prothrombin complex concentrate (median dose 2,000 IU; lower quartile 2,000, upper quartile 3,000) significantly reduced the INR from 1.7 +/- 0.1 at baseline to 1.4 +/- 0.1 (*P *< 0.001). This decrease was unrelated to fresh frozen plasma or vitamin K administration. Bleeding stopped after prothrombin complex concentrate administration in 4/11 (36%) patients with surgical bleeding and 26/27 (96%) patients with diffuse bleeding. Hemoglobin levels increased significantly from baseline in bleeding patients (*P *< 0.05) and mean arterial pressure stabilized (*P *< 0.05). No thrombotic events or changes in organ function were reported in any patient.

**Conclusions:**

Prothrombin complex concentrate application effectively reduced international normalized ratios in anticoagulation reversal, allowing surgical procedures and interventions without major bleeding. In bleeding patients, the improvement in coagulation after prothrombin complex concentrate administration was judged to be clinically significant.

## Introduction

An increasing number of people in economically developed nations are receiving oral anticoagulants for the treatment and prophylaxis of thromboembolic diseases [1,2]. Among the most commonly used oral anticoagulants are the synthetic coumarin derivatives warfarin, acenocoumarol and phenprocoumon. All three drugs act by inhibiting the biosynthesis of the vitamin K-dependent clotting factors (factors II, VII, IX and X), which produces a functional deficit of these procoagulant proteins [2]. The main indications for vitamin K antagonists are: primary and secondary prevention of venous thromboembolism; prevention of systemic embolism (for example, stroke) in patients with mechanical heart valves or atrial fibrillation; and prophylaxis (as adjunctive therapy) for systemic embolism following myocardial infarction [3].

While the antithrombotic benefits of oral anticoagulants are well established, these therapies increase the risk of hemorrhagic events, some of which may be severe or even life-threatening [4-7]. The risk of bleeding in patients receiving anticoagulants increases with surgery, trauma, over-anticoagulation or raised international normalized ratios (INRs) - although complications can still occur when the INR is within the therapeutic range [1,2,4,5,8-10].

Because of the association between vitamin K antagonists and an increased risk of hemorrhagic events, patients undergoing emergency procedures and those with life-threatening/major bleeding or highly elevated INRs require urgent and immediate reversal of anticoagulant activity [1,5,11]. Recommended treatments for rapid reversal of oral anticoagulant therapy include fresh frozen plasma (FFP) and prothrombin complex concentrates (PCCs); in all cases these should be supplemented with oral or intravenous vitamin K [1,3,5,11-17]. PCCs, which contain three or four vitamin K-dependent clotting factors, offer a number of advantages over FFP. These include a lower volume of infusion, ambient storage and reconstitution, lack of blood group specificity, a more favorable safety profile and improved efficacy [1,9]. Because of these properties, several clinical practice guidelines now recommend PCCs, in preference or as an alternative to FFP, for rapid anticoagulant reversal [1,3,5,11-17]. The PCC used in this study (Beriplex P/N^®^, CSL Behring, Marburg, Germany) contains factors II, VII, IX and X in addition to the vitamin K-dependent coagulation inhibitors protein C and protein S [18]. Beriplex P/N^® ^is prepared using pasteurization and nanofiltration to facilitate viral inactivation and elimination [19].

As PCCs are able to normalize levels of vitamin K-dependent clotting factors, and re-establish hemostasis, they may also be used as adjunctive therapy in patients with massive bleeding. Indeed, in some European countries, including Germany, PCCs are prescribed routinely for the management of massive peri- or post-operative bleeding, even though clinical data in this setting are lacking [20,21].

The objective of this study was to retrospectively evaluate the use of PCCs for perioperative treatment in a surgical patient cohort. We examined the impact of PCC therapy on coagulation and circulatory parameters and additional blood product use, and measured whole blood hemoglobin levels and specific parameters of organ dysfunction to assess the safety profile.

## Materials and methods

The study was a retrospective analysis of case notes describing the medical history and clinical management of 50 adults admitted to the surgical department at the University of Munich Hospital between 1 January and 31 December 2004, who received an infusion of PCC. The analysis was approved by the hospital's ethical review board. No exclusion criteria were applied; all patients receiving PCC entered consecutively into the study.

Patients were subdivided into those considered by the treating clinical team to require urgent and immediate reversal of vitamin K antagonist therapy and those treated for severe bleeding. The clinical requirement for PCC therapy in the bleeding group was assessed on the basis of life-threatening bleeding as diagnosed by the physician on duty and indicated by INR >1.1. Hemoglobin levels of ≤7 g/dl triggered red blood cell (RBC) transfusions in patients without cardiac risk. In patients with cardiac risk, a transfusion trigger of ≤9 g/dl was applied. Life-threatening bleeding was defined as the loss of more than 150 ml per minute or replacement of total blood volume within three hours.

The PCC used in this study was Beriplex P/N^® ^500 U, which contains 400 to 960 international units (IU) factor II, 200 to 500 IU factor VII, 400 to 620 IU factor IX and 440 to 1,200 IU factor X. In all cases, the PCC was administered by the physician on duty. The dose of PCC therapy was determined according to baseline INR, the extent and location of any bleeding and the clinical scenario. For anticoagulation reversal, patients were treated with PCC according to a standard protocol, which is in line with recent guidelines [16,22]; the aim was to attain an INR of 1.7 prior to surgery. The PCC dose was calculated from the Quick value using the formula: target Quick value (%) - actual Quick value (%) × body weight (kg) = dose in IU. The INR targeted for anticoagulation reversal patients is higher than that targeted for bleeding patients (INR of 1.2) due to the need to balance an acceptable risk of bleeding with sufficient prevention of thromboembolism. The PCC was administered intravenously (via central or peripheral venous lines) over a 10- to 20-minute period. PCC administration was started 30 minutes prior to surgery or planned intervention in reversal patients. We have used the same procedure for many years and have found a very good relationship between the dose administered and the change in Quick value (INR). Therefore, INR was not routinely determined before starting surgery. This analysis focuses on the perioperative use of PCC (up to the first post-operative day) and does not consider the effect of PCCs given thereafter. In patients with severe bleeding, repeat doses were given if necessary. All RBC and additional procoagulant hemostatic therapies (platelets, fibrinogen concentrate, FFP, desmopressin or vitamin K) administered during the six hours before and six hours after PCC administration were recorded.

Blood (6 ml citrated, 10 ml serum and 4 ml Ethylendiamin-tetraacetat (EDTA)) was routinely drawn for determination of coagulation hemoglobin and safety parameters before application of PCC. Coagulation was evaluated using the INR and Quick value (Thromborel S, Siemens, Erlangen, Germany). The INR is the ratio of a patient's prothrombin time to a normal sample, raised to the power of the International Sensitivity Index value for the thromboplastin used. Quick value is a function of the reciprocal value of a patient's prothrombin time versus that of standard human plasma, expressed as a percentage. INR was assessed less than three hours before PCC administration (pre-treatment value) and up to three hours post-dose, when the patient had returned to the surgical ward.

Safety assessments included evaluation of hemoglobin levels and serum concentrations of bilirubin (BELT 2, Roche GmbH, Mannheim, Germany), creatinine (CREY 2, Roche GmbH, Mannheim, Germany) and C-reactive protein (CRP, CRPLX, Roche GmbH, Mannheim, Germany) C-reactive protein (CRP) and CRPLX before and three days after PCC administration. In addition, vital signs (that is, body temperature, blood pressure and heart rate) were also evaluated before and within six hours after PCC administration.

Patient data were obtained from a review of patient charts, medical records and other relevant documentation. Due to the observational nature of this analysis, there was no pre-specified primary endpoint. Study endpoints included change from baseline (pre- *vs *post-PCC treatment) in INR, and concentrations of hemoglobin, serum creatinine, serum bilirubin and CRP. Arterial pressures were measured via an arterial line using a non-invasive technique within six hours pre- and post-PCC application. Hemostatic endpoints included cessation of acute bleeding, prevention of bleeding during interventional procedures and utilization of alternative blood component replacement therapies within six hours pre- and post-PCC application. Unless otherwise specified, all data are expressed as mean ± standard error of the mean (SEM). Statistical evaluation was performed with non-parametric testing (Wilcoxon) for inter-group and intra-group comparisons taking into consideration the small number of patients and the heterogeneity in clinical treatment. Significance was defined as *P *< 0.05.

## Results

Patient demographics and baseline characteristics are shown in Table 1.

**Table 1:** Patient demographics and baseline characteristics

Variable	Reversal group (n = 12)	Bleeding group (n = 38)
Gender, n (%)		
Male	6 (50)	28 (74)
Female	6 (50)	10 (26)
		
Age, years; mean (SEM)	67.3 (4.1)	66.1 (1.8)
		
Body mass index, kg/m^2^; mean (SEM)	24.5 (1.2)	23.4 (0.8)
		
Bodyweight, kg; mean (SEM)	74.7 (4.0)	69.2 (4.7)
		
Blood pressure, mm Hg; mean (SEM)		
Systolic	114.1 (7.3)	92.1 (3.7)
Diastolic	61.4 (5.2)	48.1 (2.4)
		
Heart rate, beats/minute; mean (SEM)	102.4 (11.0)	112.9 (4.9)
		
Body temperature, °C; mean (SEM)	37.2 (0.2)	36.8 (0.3)
		
Prior history of bleeding, n (%)	4 (33)	4 (11)
		
Pre-treatment INR, mean (SEM)	2.8 (0.2)	1.7 (0.1)*
		
Pre-treatment Quick value, %; mean (SEM)	33.1 (2.7)	54.7 (2.6)*
		
Baseline laboratory assessments, mean (SEM)		
Hemoglobin, g/dl	11.8 (0.6)	8.2 (0.3)*
Serum creatinine, mg/dl	2.0 (0.5)	1.3 (0.2)
Serum bilirubin, mg/dl	3.3 (1.1)	2.3 (1.2)
C-reactive protein, mg/dl	8.7 (4.1)	9.4 (1.8)
		
Indication for vitamin K antagonist therapy, n (%)		
Atrial fibrillation	4 (33)	N/A
Heart valve replacement	3 (25)	N/A
Pulmonary embolism	1 (8)	N/A
Thrombosis prophylaxis	1 (8)	N/A
Post-myocardial infarction	3 (25)	N/A
		
Use of low-molecular weight		
heparin, n (%)	2 (17)	25 (66)

* *P *< 0.001

INR = international normalized ratio; N/A = not applicable; SEM = standard error of the mean

### Patients requiring urgent reversal of oral anticoagulation

Of the 12 patients who required urgent reversal of oral anticoagulation, the majority were receiving prophylactic vitamin K antagonist therapy (intravenously) following atrial fibrillation (n = 4) or mechanical heart valve replacement (n = 3). Two patients were also receiving concomitant low-molecular weight heparin as bridging therapy before a planned intervention.

The indications for PCC treatment in this group of patients included: emergency surgery (vascular [n = 2], trauma [n = 2] and abdominal [n = 1] surgery); post-trauma (intracranial [n = 1] and intramuscular [n = 1] hemorrhage); cholecystitis [n = 1]; bleeding due to rectal cancer [n = 1]; endoscopic intervention [n = 1]; and coagulation failure (during emergency [n = 1] and trauma [n = 1] surgery) (Table 2). Two of the patients - one with cholangitis and one with intracranial bleeding - did not undergo an invasive procedure.

**Table 2:** Distribution of patients in anticoagulation reversal and bleeding groups by discipline

Discipline	Reversal group (n = 12)	Bleeding group (n = 38)
General surgery	7 (58%)	30 (79%)

Vascular surgery	1 (8%)	6 (16%)

Trauma	2 (17%)	2 (5%)

Conservative therapy	2 (17%)	0 (0%)

**Figure 1:**
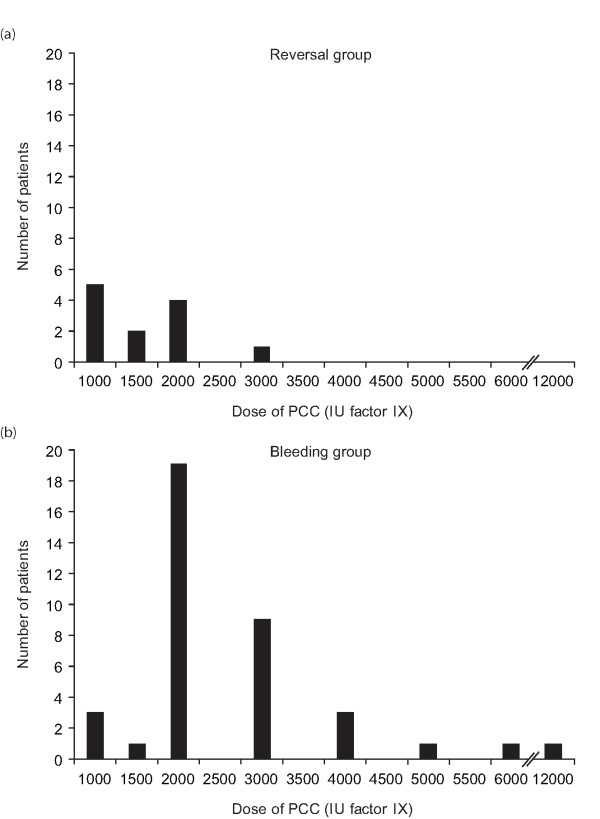
Dose of prothrombin complex concentrate administered to: (a) surgical patients requiring urgent reversal of vitamin K antagonist therapy, (b) patients with severe bleeding.

**Figure 2:**
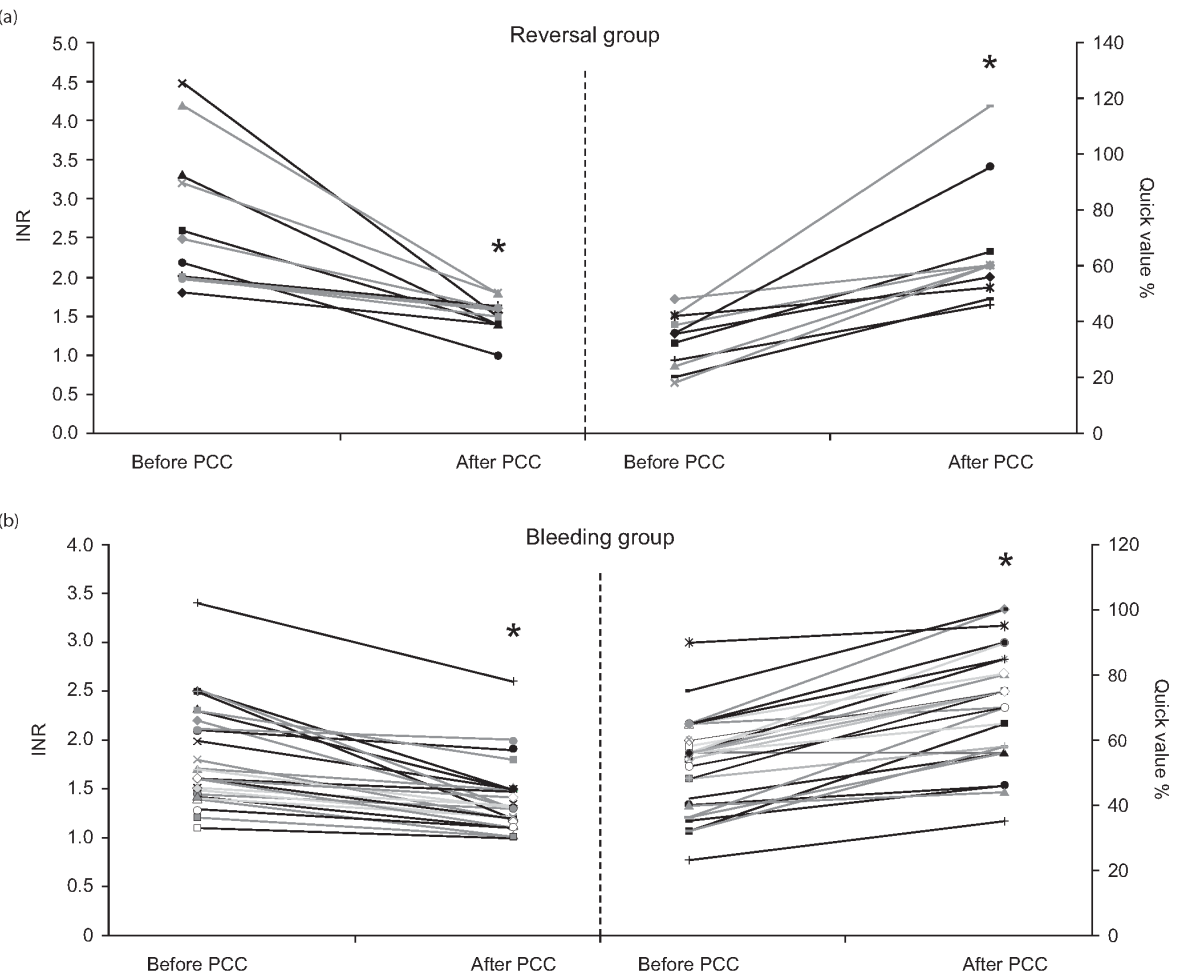
International normalized ratios and Quick values (%) before and after infusion of prothrombin complex concentrate in: **(a) **patients requiring urgent reversal of vitamin K antagonist therapy; and **(b) **patients with severe bleeding**.** * P < 0.001 *vs *before in prothrombin complex concentrate.

The median dose of PCC administered was 1,500 IU (lower quartile 1,000, upper quartile 2,000 IU; Figure 1a). The mean INR decreased significantly (*P *< 0.001) from 2.8 ± 0.2 at baseline to 1.5 ± 0.1 at 180 ± 31 minutes (the mean time of the first INR measurement after PCC administration; Figure 2a). There was a corresponding significant increase in Quick values (%) from 33.0 ± 2.9 at baseline to 65.4 ± 6.5; *P *< 0.001 (Figure 2a). The most common additional conservative therapy, either before or after PCC, was intravenous vitamin K (administered before PCC on four occasions and after on three occasions; Table 3). The mean dose of vitamin K administered was 21 ± 4 mg (i.v.). Vitamin K was not routinely administered by the physician on duty when the operative procedure was to be performed within four hours. Two patients received platelets, RBCs and FFP, either before or after PCC.

**Table 3:** Patients administered hemostatic therapies and allogeneic blood component transfusions in conjunction with prothrombin complex concentrate

	Number of patients (%)			
	
	Reversal group (n = 12)		Bleeding group (n = 38)	
**Hemostatic therapies**				
				
Vitamin K	7 (58)		5 (13)	

Desmopressin	0 (0)		7 (18)	

Fibrinogen concentrate	0 (0)		4 (11)	

**Allogeneic blood components**	**Within 6 h before PCC**	**Within 6 h after PCC**	**Within 6 h before PCC**	**Within 6 h after PCC**

Red blood cells	1 (8)	1 (8)	21 (55)	16 (42)

Fresh frozen plasma	0 (0)	2 (17)	12 (32)	11 (29)

Platelets	1 (8)	1 (8)	7 (18)	3 (8)

PCC = prothrombin complex concentrate.

No major perioperative bleeding was reported in anticoagulation reversal patients following PCC infusion. Moreover, prophylactic PCC application allowed operative and interventional procedures to be performed without the need for blood component replacement therapy in all but two of the patients.

Three days after PCC administration, serum creatinine and bilirubin concentrations were not significantly increased, but CRP was significantly higher than baseline (*P *< 0.05), as expected after an intervention or operation. Hemoglobin concentrations were comparable before and after PCC treatment.

### Patients treated for severe bleeding

None of the 38 patients treated for severe bleeding were receiving coumarin derivatives at the time of treatment; before the bleeding episode, two were receiving aspirin and 25 were receiving low molecular weight heparin as low-dose thromboprophylaxis. Thirty patients (79%) were undergoing general surgery, six (16%) vascular surgery, and two (5%) required surgery as a result of trauma (Table 2). The different locations of bleeding are summarized in Table 4.

**Table 4:** Bleed location or cause in patients with severe bleeding

Location/cause of bleeding	Number of patients (%)
Unknown	8 (21%)

Liver parenchyma	6 (16%)

Diffuse abdominal	6 (16%)

Upper gastrointestinal tract	5 (13%)

Spleen	3 (8%)

Retroperitoneal	3 (8%)

Lower gastrointestinal tract	2 (5%)

Mucosal	1 (3%)

Bleeding after aneurysm operation	1 (3%)

Arrosion bleeding from the hepatic artery	1 (3%)

Bronchial	1 (3%)

Extremity	1 (3%)

The median dose of PCC administered was 2,000 IU (lower quartile 2,000, upper quartile 3,000 IU; Figure 1b). In one patient with an abdominal gun-shot wound associated with massive retroperitoneal bleeding, a bolus of 6,000 IU of PCC was applied followed by a continuous infusion with 1,000 IU/hour to a total dose of 12,000 IU as post-operative bleeding did not stop despite extensive surgical procedures including nephrectomy, liver resection and abdominal packing. In bleeding patients, administration of PCC resulted in a significant reduction (*P *< 0.001) in the mean INR (from 1.7 ± 0.1 at baseline to 1.4 ± 0.1; Figure 2b), 147 ± 15 minutes after treatment (the mean time of the first INR measurement). Mean Quick values (%) also increased significantly (*P *< 0.001) from 53.4 ± 2.3 at baseline to 72.1 ± 2.7 (Figure 2b). Bleeding stopped after administration of PCC in 4 of 11 (36%) patients with surgical bleeding (i.e. bleeding associated with vascular damage that can rarely be controlled without revision surgery). In patients with diffuse bleeding (that is, pure, *oozing *tissue bleeding with no evidence of damaged blood vessels), the active bleeding stopped after PCC therapy in 26 of 27 (96%) affected patients.

Additional conservative therapies administered to 27 patients within six hours before and 22 patients within six hours after PCC in the bleeding group were (in descending order of frequency): RBC, FFP, platelets, desmopressin, intravenous vitamin K and fibrinogen concentrate (Table 3). Twenty of the 22 patients given additional conservative therapies within six hours after PCC administration received allogeneic blood components: RBC only (n = 8); RBC and FFP (n = 5); FFP only (n = 4); RBC, FFP and platelets (n = 2); RBC and platelets (n = 1).

**Figure 3:**
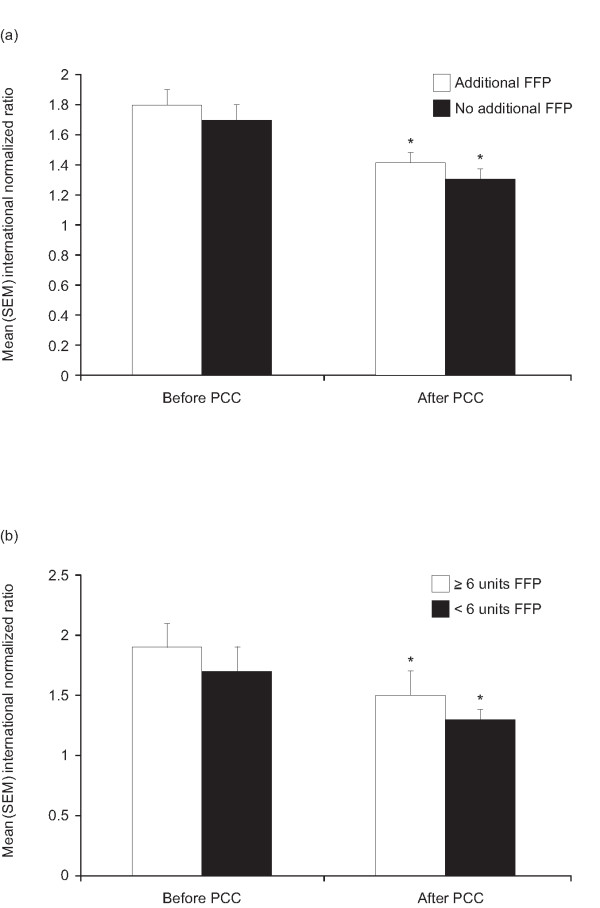
Mean ± standard error of the mean international normalized ratios before and after infusion of prothrombin complex concentrate in patients with severe bleeding. **(a) **Additional FFP treatment did not influence INR, white bars: patients receiving additional fresh frozen plasma (FFP) (n = 11); black bars: patients not receiving additional FFP (n = 27). **(b) **Administration of more units of FFP did not influence INR, white bars: patients receiving ≥ 6 units additional FFP (n = 4); black bars: patients receiving <6 units additional FFP (n = 7). * *P *< 0.001 *vs *pre-PCC.

**Figure 4:**
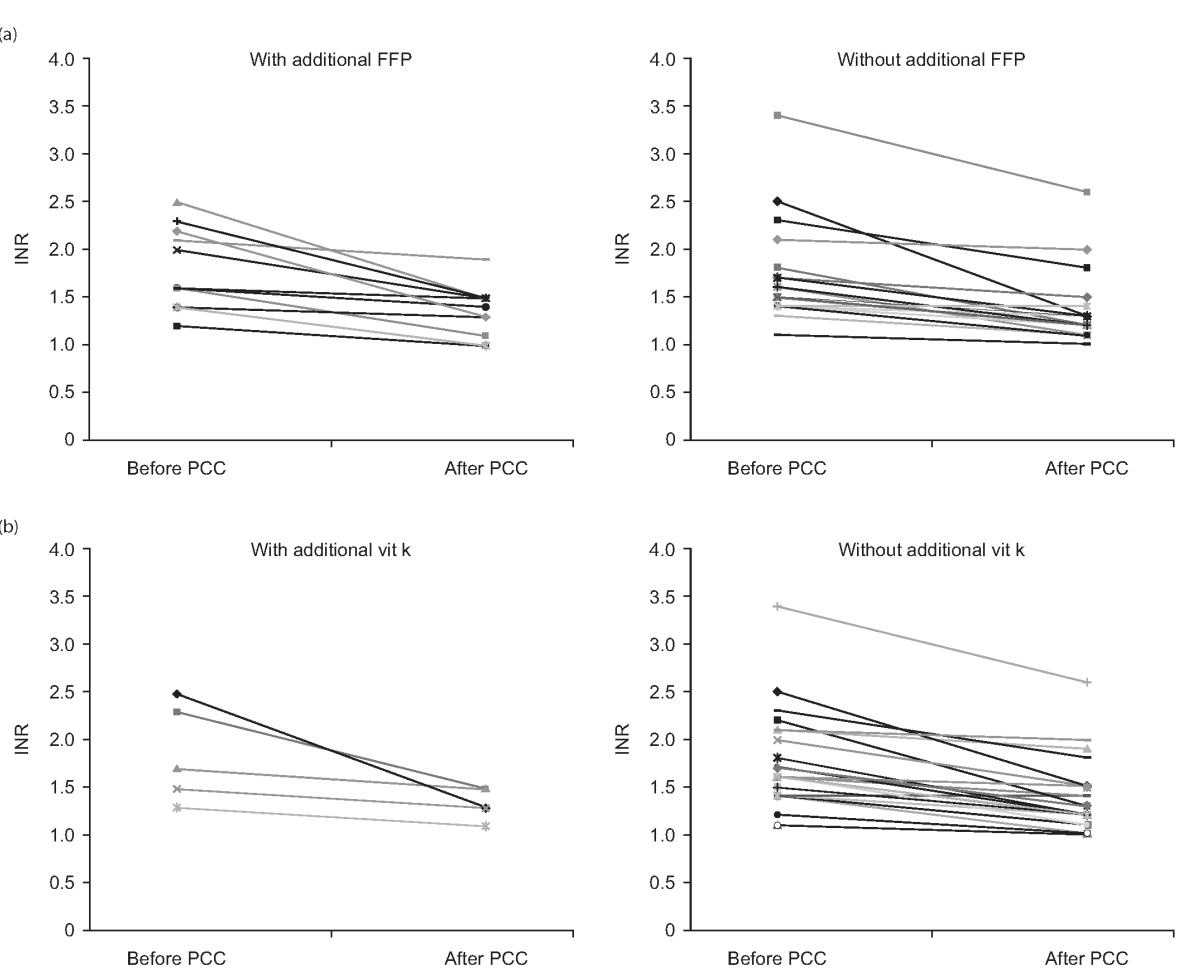
International normalized ratios before and after infusion of prothrombin complex concentrate in patients with severe bleeding who received additional conservative therapies: the change in INR was unaffected by the addition of (a) FFP, n = 11 and (b) vitamin K, n = 5.

The significant reduction in INR observed in the bleeding patients was unrelated to whether or not patients received FFP or vitamin K between sampling for measurement of the baseline INR and the INR attained (Figure 3a). Among those patients receiving FFP, there was also no significant difference in reduction of INR between patients receiving less than six units and those receiving six units or more (Figure 3b). Additional conservative therapies did not modify the effect of PCC on INR or Quick values in this patient group (Figure 4a and 4b). Also, when comparing the mean percentage change in INR from baseline values, no difference was detected between patients receiving FFP (with FFP: 23.0 ± 4.0%; without FFP: 13.5 ± 2.1%; *P *= 0.39) or vitamin K (with vitamin K: 24.7 ± 7.0%; without vitamin K: 13.7 ± 1.8%; *P *= 0.35).

**Figure 5:**
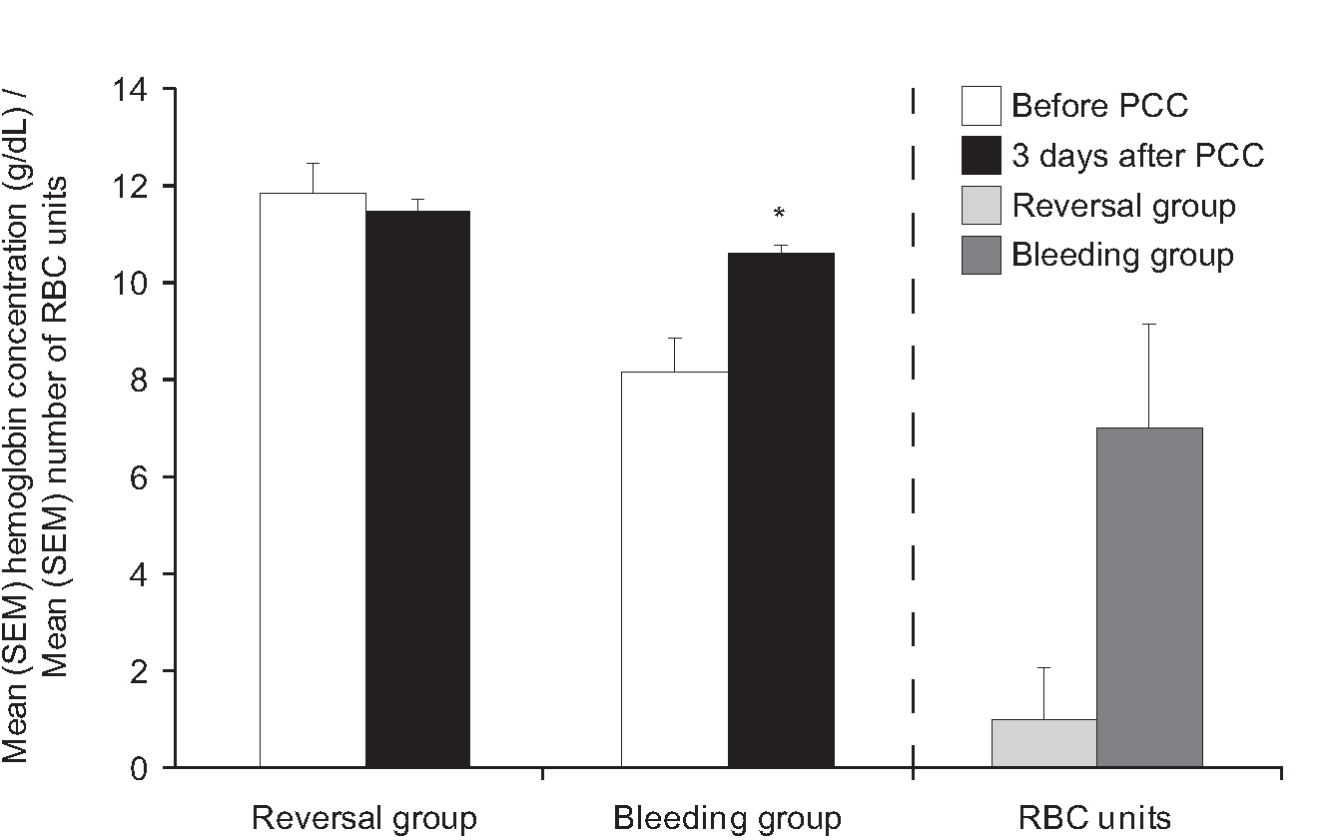
Mean ± standard error of the mean hemoglobin concentrations in patients requiring urgent reversal of vitamin K antagonist therapy (reversal) or with severe bleeding (bleeding). White bars: before (baseline); black bars: after infusion of prothrombin complex concentrate. The mean ± SEM units of red blood cells transfused in each patient group are also shown. Light gray bar: patients requiring urgent reversal of vitamin K antagonist therapy; dark gray bar: patients with severe bleeding. * *P *< 0.05 *vs *baseline.

**Figure 6:**
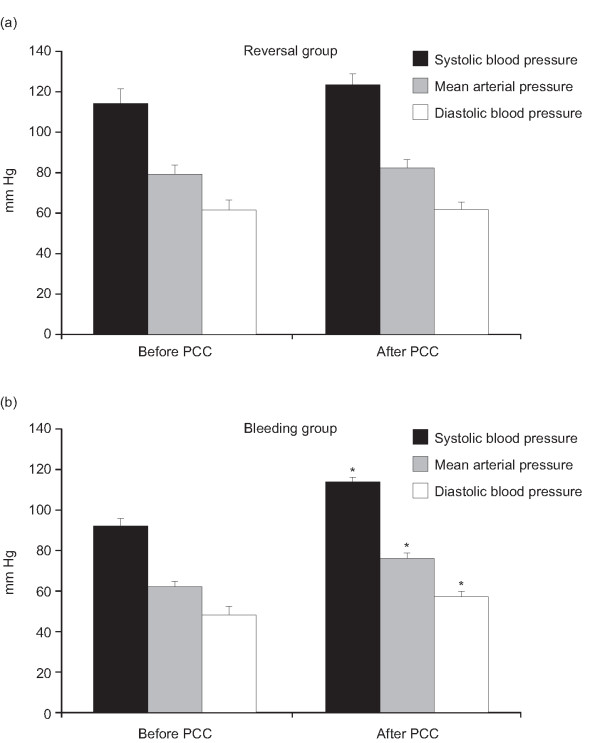
Mean ± standard error of the mean systolic and diastolic arterial blood pressure in patients requiring urgent reversal of vitamin K antagonist therapy (reversal) or with severe bleeding (bleeding). Black bars: systolic blood pressure; gray bars: mean arterial pressure; white bars: diastolic blood pressure. * *P *< 0.05 *vs *before prothrombin complex concentrate.

**Figure 7:**
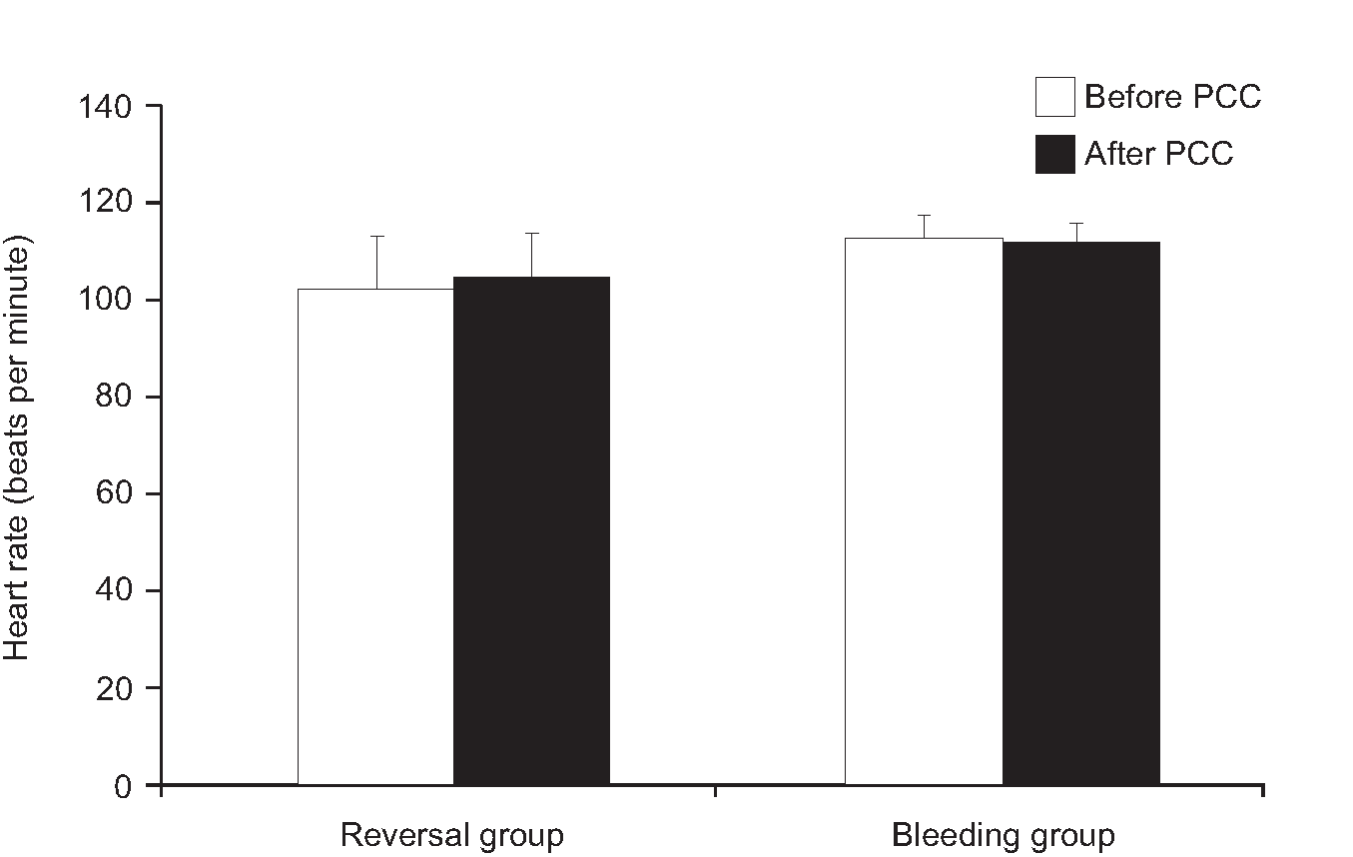
Mean ± standard error of the mean heart rate in patients requiring urgent reversal of vitamin K antagonist therapy (reversal) or with severe bleeding (bleeding). There was a significant increase in systemic blood pressure in bleeding patients after prothrombin complex concentrate application (*P *< 0.01 *v*s before PCC). White bars: before (baseline); black bars: after infusion of prothrombin complex concentrate (PCC).

Hemoglobin levels increased significantly (*P *< 0.05) from 8.2 ± 0.3 g/dl at baseline to 10.6 ± 0.2 g/dl after PCC treatment (Figure 5) although a comparable amount of RBC was applied within six hours before and after PCC treatment (Table 3). This finding also indicates cessation of bleeding. The mean number of RBC units administered to bleeding patients was 6.9 ± 2.1, compared with one unit in one anticoagulation reversal patient (Figure 5). After administration of PCC in bleeding patients arterial pressure increased (Figure 6), whereas heart rate was unchanged (Figure 7), indicating hemodynamic stabilization. Serum creatinine and bilirubin concentrations measured three days after administration of PCC were not significantly increased. An increase in CRP was observed, but this was not statistically significant.

### Comparison of anticoagulation reversal and bleeding patients

The patient groups were comparable with regard to age and body temperature. No patient was hypothermic immediately before PCC administration (Table 1) or after treatment, when the mean (± SEM) temperature was 37.0°C (± 0.2) in both groups. The mean INR prior to PCC infusion was significantly lower in the bleeding group than in the anticoagulation reversal group (*P *< 0.001) (Table 1). In terms of safety assessments, baseline hemoglobin levels were significantly lower in bleeding patients than in anticoagulation reversal patients (*P *< 0.001), which underlies the higher rate of RBC transfusion in the bleeding patients. Baseline serum bilirubin and creatinine concentrations were also lower, although not significantly so, in the bleeding group compared with the anticoagulation reversal group. Baseline CRP concentrations were similar in the two groups. The mean dose of PCC administered was significantly higher in bleeding patients than in patients requiring anticoagulation reversal (*P *< 0.05) (Figure 1) and overall, bleeding patients received more hemostatic therapies and allogeneic blood components than anticoagulation reversal patients.

No thrombotic events or viral transmissions were reported for any of the patients during the period of hospitalization. However, there was no standard protocol in place to track potential virus transmission.

## Discussion

PCCs are recommended in various guidelines for the emergency reversal of oral anticoagulation therapy, particularly in the presence of major bleeding and/or elevated INR [1,3,5,11-17]. Despite these recommendations, the use of PCC remains low in many surgical units where emergency physicians continue to use human plasma because of its widespread availability, its low cost, its reasonable efficacy and lack of awareness of the guidelines [1,9,23]. In contrast, PCC has been used for several years in our surgical unit, since before the introduction of recent guidelines recommending its use, both for anticoagulation reversal and for adjunctive treatment of acute hemorrhage, due to its high clinical efficacy.

PCCs may offer advantages over FFP for urgent reversal of oral anticoagulation therapy [24]. Comparative studies have suggested that PCCs may provide more effective and rapid correction of INR than FFP, with a greater increase in clotting factors [25-28]. PCCs can also be infused faster than human plasma, have a reduced volume of administration (and therefore, unlike FFP, are not associated with volume overload), are associated with a shorter preparation time (since some PCCs can be stored and reconstituted at room temperature, whereas FFP needs to be thawed prior to use), and do not require blood-group matching [1].

In this study, PCC effectively normalized the INR in patients requiring emergency reversal of anticoagulation therapy. Moreover, the effect on the INR was similar to that reported in other studies in which PCCs (including Beriplex P/N^®^) had been administered with vitamin K to reverse over-anticoagulation, treat anticoagulant-related bleeding, prepare anticoagulant-treated patients for emergency surgery, or manage other miscellaneous conditions in patients receiving oral anticoagulants [8,25-27,29-36]. In particular, the relatively low dose of PCC (about 22 IU/kg) used here in the anticoagulant reversal group (baseline INR 2.8) is comparable with that used by Pabinger et al. during a recent prospective trial [30], in which 93% of emergency coagulant reversal patients with baseline INR 2 to 3.9 achieved INR ≤1.3 after receiving 25 IU/kg of the same PCC. The mean INR of 1.5 achieved with PCC administration in anticoagulation reversal patients was below the target threshold of 1.7. PCC infusion also facilitated surgical procedures in this group, as evidenced by the absence of major perioperative bleeding and low use of concomitant blood component replacement therapy. Our data add further to the evidence for using PCC in vitamin K antagonist reversal at a time when guidelines continue to discuss this issue and PCC is still not available in many European centers.

PCC also restored the INR to nearly normal values (mean of 1.4 compared with a target of 1.2) in patients with acute severe bleeding. Furthermore, the bleeding episodes ceased within three hours of PCC administration in 30 out of 38 (79%) patients (4/11 patients with surgical bleeding and 26/27 patients with diffuse bleeding). This is a particularly important observation, as clinical data demonstrating the efficacy of PCC in patients with severe bleeding not associated with oral anticoagulation therapy are scarce, despite reports dating back more than a decade of their potential and their routine use in massive hemorrhage in many European countries [20,21,37]. In a recent observational study conducted within a UK tertiary hospital, significant improvements in clotting times were reported with PCC in all but two of 20 patients with life-threatening bleeding [38]. Only five of these patients were receiving oral anticoagulants; the remaining patients had severe perioperative or post-operative bleeds. Further expansion of this retrospective review demonstrated a considerable reduction in administration of other blood products during the 24 hours following PCC administration with partial or complete hemostasis achieved in 14 of 18 cases (78%) [39]. Moreover, 83% of these patients received PCC at a lower dose (≤1,500 IU) than we used in the present study, so it is also an economically viable option.

The retrospective nature of the present study results in limitations, most notably the lack of a control group. Patients received conservative therapies in addition to PCC, and the contribution of these to the reversal of anticoagulation or cessation of bleeding cannot be ruled out. Also no blood sample of prothrombin was drawn immediately after PCC administration, reflecting that these data were collected in a real-life clinical situation, rather than being part of a prospective clinical trial. However, controlled studies are difficult to conduct in this setting - in particular in the situation where patients are suffering considerable blood loss, it would be unethical to include a control group receiving no hemostatic therapy. Additional units of FFP administered to bleeding patients were unlikely to be responsible for the increase in Quick results observed. INR values measured at baseline or after PCC administration were not significantly different between patients who did or did not receive FFP between the baseline and after PCC time points. Similarly, other additional conservative therapies, including intravenous vitamin K, were administered to some but not all patients, based on our experience with PCCs in emergency surgical patients over many years. Furthermore, intravenous administration of vitamin K would not be expected to have an impact within this four-hour period. No significant effects of these additional treatments on INR were seen in any patient group.

Another alternative to FFP for treatment of life-threatening bleeding is activated recombinant factor VII (rFVIIa). This is approved for use as a *bypassing agent *in hemophiliac patients who have inhibitor antibodies to factor VIII or IX, but its use in a broader range of applications in life-threatening hemorrhage has been extensively reported [40]. However, rFVIIa only replaces a single factor, and its principle mechanisms of action are dependent on adequate levels of other coagulation factors, in particular factors II and X, and fibrinogen [24].

The data obtained in this study do not provide information on the speed of INR correction following administration of PCC; they merely represent clotting measurements at a specific time in a given setting (mainly after surgery has been performed and the patient has been transferred to the surgical ward for post-surgical care). However, the data do suggest that correction of INR and cessation of bleeding can be achieved with PCC in less than three hours. In a recent pharmacokinetic study, administration of PCC (Beriplex P/N^®^; dose 50 IU factor IX/kg) to 15 healthy volunteers resulted in a maximal increase in coagulation factors II, VII, IX and X within five minutes of administration, suggesting that PCC has a rapid onset of action [41]. This increase in clotting factors remained stable over the next six hours and declined slowly over the next six days. One factor that will influence the speed of INR correction with PCCs is body temperature. Hypothermia inhibits the synthesis of fibrinogen and the initiation phase of thrombin generation [42] so normothermia is important for effective hemostasis [42]; all patients were normothermic at the beginning of PCC application in our study.

The magnitude of INR reduction in bleeding patients was not as large as that seen in the anticoagulation reversal group. This is likely to be due largely to a lower baseline INR in the bleeding group (1.7 *vs *2.8 in the anticoagulation reversal group), but it could also be attributed to a higher volume application in the reversal patients, different consumption of coagulation factors or cardiocirculatory instability following activation of coagulation.

The dose of PCC administered was significantly higher in bleeding patients than in anticoagulation reversal patients. This was probably because bleeding patients were incurring major blood loss, whereas anticoagulation reversal patients were only receiving PCC as bleeding prophylaxis. Therefore, higher doses were administered in an attempt to control a more urgent and immediate clinical situation. As described above, the target INR in bleeding patients was also lower than in the anticoagulation reversal patients, necessitating higher PCC doses in an attempt to achieve the lower target.

Measurements of serum creatinine and bilirubin did not suggest any detrimental effects of PCCs on kidney or liver function. With the exception of hemoglobin in bleeding patients and CRP in reversal patients, laboratory safety parameters were not increased from baseline values following infusion of PCC. The increase in hemoglobin concentrations in the bleeding group may be explained by the higher and more frequent use of RBC concentrates compared with the anticoagulation reversal group and by the eventual cessation of bleeding in these patients. Unsurprisingly, baseline hemoglobin levels were lower in bleeding patients due to the severe blood loss incurred. Baseline serum creatinine and bilirubin concentrations were also lower in bleeding patients, most likely for the same reason. The significant increase of CRP concentration in the anticoagulation reversal patients is probably due to the operative procedure that followed optimization of coagulation. In contrast, bleeding patients did not show an increase in CRP after PCC application, despite receiving higher doses of PCC on average. It therefore seems highly unlikely that PCCs induce inflammatory activation.

Although there is a small, and to a certain degree inherent, risk of thromboembolic events when PCCs are used for anticoagulation reversal [2,9,20], there was no evidence of any thromboembolic complications in this study. There was also no evidence of viral transmission, something that is very rarely reported with PCC therapy, in any of the patients in this study [2]. Effective virus inactivation and reduction processes are reported for the study drug, which make transmission of infective agents, including prions, less likely [19]. These results and those reported in other similar studies suggest that Beriplex P/N^® ^has a favorable safety profile with a low risk of thromboembolic complications [8,30,31,35].

## Conclusions

PCCs can effectively improve INR in non-hypothermic surgical patients requiring vitamin K antagonist reversal or those experiencing severe bleeding. In almost all patients, the improvement in coagulation was judged to be clinically significant, and allowed operative and/or interventional procedures to be performed. Thus, PCC application in anticoagulation reversal and bleeding surgical patients appears to be effective with a favorable safety profile and, as such, warrants further prospective evaluation.

## Key messages

• This study shows that PCC therapy effectively reduces INR among patients requiring urgent vitamin K antagonist reversal, and those with severe bleeding.

• The improvement in coagulation produced by low doses of PCC was clinically significant, and allowed operative and/or interventional procedures to be performed without major bleeding.

• The use of PCC among patients with severe bleeding is novel and warrants further evaluation.

• The safety of PCC therapy was favorable in this study, with no thrombotic events or changes in organ function reported in any patient.

## Abbreviations

CRP: C-reactive protein; FFP: fresh frozen plasma; INR: international normalized ratio; IU: international units; PCC: prothrombin complex concentrate; RBC: red blood cells; SEM: standard error of the mean.

## Competing interests

This study was supported by a restricted grant from CSL Behring, Marburg, Germany. Prof. Johannes N. Hoffmann has received funding from CSL Behring, Biotest, Roche and Octapharma. Dr Kerstin S. Schick, Dr Jan M. Fertmann and Prof. Karl-Walter Jauch declare no funding.

## Authors' contributions

KSS contributed to data acquisition and interpretation and reviewed the manuscript. JMF contributed to data acquisition and reviewed the manuscript. K-WJ contributed to data interpretation and reviewed the manuscript. JNH conceived of the study, participated in its design and coordination, and contributed to data acquisition and interpretation and writing and reviewing the manuscript. All authors read and approved the final manuscript.
